# Performance Evaluation of an Infrared Thermocouple

**DOI:** 10.3390/s101110081

**Published:** 2010-11-10

**Authors:** Chiachung Chen, Yu-Kai Weng, Te-Ching Shen

**Affiliations:** 1 Department of Bio-industrial Mechatronics Engineering, National ChungHsing University, 250 Kuokuang Road, Taichung, Taiwan; 2 Department of Biomechatronics Engineering, National Chia-I University, Chia-I, Taiwan

**Keywords:** infrared thermocouple, thermometer, calibration equation, leaf temperature

## Abstract

The measurement of the leaf temperature of forests or agricultural plants is an important technique for the monitoring of the physiological state of crops. The infrared thermometer is a convenient device due to its fast response and nondestructive measurement technique. Nowadays, a novel infrared thermocouple, developed with the same measurement principle of the infrared thermometer but using a different detector, has been commercialized for non-contact temperature measurement. The performances of two-kinds of infrared thermocouples were evaluated in this study. The standard temperature was maintained by a temperature calibrator and a special black cavity device. The results indicated that both types of infrared thermocouples had good precision. The error distribution ranged from −1.8 °C to 18 °C as the reading values served as the true values. Within the range from 13 °C to 37 °C, the adequate calibration equations were the high-order polynomial equations. Within the narrower range from 20 °C to 35 °C, the adequate equation was a linear equation for one sensor and a two-order polynomial equation for the other sensor. The accuracy of the two kinds of infrared thermocouple was improved by nearly 0.4 °C with the calibration equations. These devices could serve as mobile monitoring tools for *in situ* and real time routine estimation of leaf temperatures.

## 1. Introduction

Monitoring of the physiological state of forests or agricultural plants has become a basic technique to control the crop environment and to modulate irrigation and fertilization [1]. Remote and non-destructive monitoring techniques are more convenient to use to collect the necessary physiological information, including important factors such as plant weights, stem diameters, sap flow rates, fruit diameters and leaf temperatures.

Jones [2] introduced an infrared thermometry method to detect stomatal closure conditions. Artificial wet and dry reference surfaces were used to provide the base indexes. Kumar *et al*. [3] applied a Tele temp AG-42 infrared thermometer to measure the canopy temperature and used these measurement values as an indicator of plant-water status. Gontia and Tiwari [4] selected an infrared thermometer to measure the leaf temperature of wheat crops. The difference in the canopy and air temperature and the vapour pressure deficit was used to calculate the crop water stress index.

The performance evaluation of the infrared thermometer is a key step to ensure the accuracy of the measured values. Amiro *et al*. [5] described the design of leaf chambers and proposed a nonlinear calibration equation. The accuracy of measurement values of their infrared thermometer was limited to within 0.2 °C. Rapier and Michael [6] introduced a calibration technique for Everest 4000A Radiometers. A temperature control chamber was constructed for the calibration equipment and a polynomial calibration equation was established. Their results revealed that the root-mean-square differences were less than 0.6 °C.

Kalma and Alksnis [7] described a calibration system for an infrared surface temperature transducer. The observed significant measurement errors were due to the assumption of a linear relationship between the output voltage and standard values detected by a thermistor thermometer. Bugbee *et al*. [8] evaluated two types of infrared transducers and found that the need for a longer response period to reach the equilibium state induced significant errors. The best calibrations for these infrared thermometers were second-order polynomial equations. Baker *et al*. [9] tested the accuracy of an infrared thermometer using the measurement values of a calibrated thermocouple as the standard values. Their results showed the mean absolute error of this infrared thermometer was 0.04 °C for measurement values higher than 24 °C. Savage and Heilman [10] selected a shortwave calibrator to test the performance of twenty-one infrared thermometers. A third-order polynomial equation was proposed to express the relationship between the measurement values of the infrared thermometers and standard values obtained from the calibrator. All residual errors were within 0.15 °C.

The quantitative sensor performance criteria are very important to assess the accuracy and precision. The regression analysis of the linear equation or the higher-order polynomial equation of the relationship between the readout values of an instrument and the standard values of the calibrator is the basic statistical technique used to evaluate performance. The indexes of slope, intercept and the standard deviation are usually selected to evaluate the performance [11–13]. Some criteria, derived from the difference between measurement values and standard values, have been adopted by other researchers [10].

The components of an infrared thermometer include an optical system to collect the energy emitted by the measurement subject, the lens to screen the specific wavelength, a detector to convert the energy to an electrical signal, an emittivity adjustment, an ambient temperature compensation circuit and the microprocessor-based electronics to calculate the temperature according to some pre-set equation. For the infrared thermometer, the received energy is detected and converted into a voltage signal using a pyroelectric or thermopile device. This device requires an external power supply and they are expensive.

A novel self-powered infrared thermocouple has been recently developed by some manufacturers of infrared thermometers. This device can detect the radiation energy and imitate it as a thermocouple output. Because of the simplicity and compatibility with the thermocouple’s transducer, these units are inexpensive. The limitation is the measuring range [11]. Within the narrow temperature range, the output signal has a linear relationship with the target temperature. The radiation energy is converted into an electrical potential by a preset linear equation. To evaluate the affordability of the two types of devices—infrared thermometer and infrared thermocouple—Mahan and Yeater [14] compared their performance and determined their reliability in field tests. Their results indicated that the traditional infrared thermometer agreed more closely with the reference temperature than that measured with a thermocouple wire. The detected leaf temperatures for grass cotton over several days showed that the two types of device gave similar measurement values over a 13–35 °C range.

Recently, more types of infrared thermocouples have been commercialized by manufacturers. This provides an opportunity to apply the technique for monitoring routine temperatures for forest management and agricultural production. The objectives of this study were: (1) to evaluate the performance of infrared thermocouples using a standard temperature calibrator and (2) to determine the adequate calibration equations to improve the accuracy of these sensors.

## 2. Materials and Methods

### 2.1. Infrared Thermocouple Devices

Two kinds of infrared thermocouples were used in this study, a Sentron SI-10AL (Sentron Eng. CO, Ltd, Taiwan) and a Trotec BP-20 (Trotec GmbH & CO, Heinsberg, Germany). The respective manufacturer’s specifications are listed in Table 1.

### 2.2. Standard Temperature

The standard temperature of the blackbody source was maintained by a TC 2000 temperature calibrator (Instutek AS, Skreppestad Naringspak, Norway). The operating temperature ranged from −40 °C to 150 °C. The temperature of the standard environment was measured by a RTD (resistive temperature detector) thermometer calibrated by the U.S. NIST (National Institute of Standards and Technology). The uncertainty of this equipment from the calibration certificate was 0.03 °C. An aluminum cylinder was installed into the oil bath of this calibrator. The size of this cylinder corresponded to the requirements of the blackbody source [15].

### 2.3. Test Procedures

The target temperature for calibration was maintained at 13, 17, 21, 25, 29, 33 and 37 °C for the Sentron SI-10A and at 2 °C intervals for the Trotec BP-20 within the same measuring range. The test environment was maintained at 25 °C and the variation of the set room environment temperature was controlled within ±1.5 °C. Several replicate measurements were made for each standard temperature. As one measurement has finished, the infrared thermocouple was taken out from the blackbody cavity for five minutes and then was put into the cavity for further measurement. The signals of the two kinds of infrared thermocouples were indicated in their LCD screens. The error of the data acquisition device was insignificant, according to the manufacturer’s specifications. The sensor signals reached an equilibration state within one second and this was recorded by the visual method.

### 2.4. Data Analysis

The performance of these infrared thermocouples was assessed by their accuracy and precision. The accuracy is expressed as the closeness with which a measurement value approached the standard temperature, *i.e.*:

(1) $$e_{i}=\;T_{r}-\;T_{s}$$

where *e_i_* is the error, *T_r_* is the readout value of infrared thermocouple, and *T_s_* is the standard temperature. The smaller the *e_i_* value, the better the accuracy.

The precision *P* is expressed as the repeatability of these measurement values in the same standard environment. The precision P is exactly the statistical standard deviation computed from the available set of temperature measurements:

(2) $$P={\left[\left(\frac{\sum {\left(T_{r}-T_{\mathrm{ave}}\right)}^{2}}{n-1}\right)\right]}^{0.5}$$

where *T_ave_* is the average value of reading values and *n* is the number of data. A smaller *P* value indicates better precision performance.

### 2.5. The Calibration Equation

The calibration equations were established using the regression analysis technique. The criteria for selecting of the best equation are the coefficient of determination *R^2^*, the standard deviations of estimator, *s*, for the calibration equation, the t-test of each parameter for the equation, and residual plots [16]. The R^2^ value is calculated as follows:

(3) $$R^{2}=\frac{\sum {\left(y\prime -y_{\mathrm{ave}}\right)}^{2}}{\sum {\left(y_{i}-y_{\mathrm{ave}}\right)}^{2}}$$

where y’ is the predicted value of the regression equation, y_ave_ is the average value of the dependent value and y_i_ is the the dependent value.

(4) $$s^{2}=\frac{\sum {\left(y_{i}-y^{\prime }\right)}^{2}}{n-p}$$

where n is the number of data and p is the number of parameters of equation.

The t value of each parameter is calculated as:

(5) $$t=b_{i}/\text{se}\left(b_{i}\right)$$

where b_i_ is the parameter values and se(b_i_) is the standard estimation values of b_i_.

The residual plot is a qualitative criterion to evaluate the adequateness of calibration equations. As the data distribution of residual plots indicated a uniform pattern, the model could be recognized as an adequate model. If the residual plots revealed a clearly systematic pattern, a model cannot be accepted.

There are two types of calibration equations. For the classical calibration equation, the readout values of infrared thermocouples were assumed as dependent variables and the standard values of the calibrator were selected as independent variables. For the inverse calibration equation, the standard values maintained by the temperature calibrator were recognized as dependent variables. Because the inverse calibration equation has better predictive ability and is easy to apply [12,13], the inverse calibration equation was adopted in this study:

(4) $$T_{s}=b_{o}+T_{r}+b_{2}T_{r}^{2}+b_{3}T_{r}^{3}+......+b_{n}T_{r}^{n}$$

where *b_o_, b_1_, b_2_*, …. *b_n_* are parameters.

A polynomial equation could be used to fit the calibration data. However, as higher order parameters were selected, the predicted errors could be increased significantly [16], so the selection of an adequate calibration equation is very important for the sensor performance. The selection of the optimum b_o_, b_i_ to b_n_ in Equation (4) was based on the statistical criteria, t-test values of each parameter and residual plots. The procedures were executed by the software SigmaPlot ver. 10.0 (Systat software Inc, IL, USA).

## 3. Results and Discussion

### 3.1. Performance of the Sentron SI-10A Sensor

**Figure 1.**
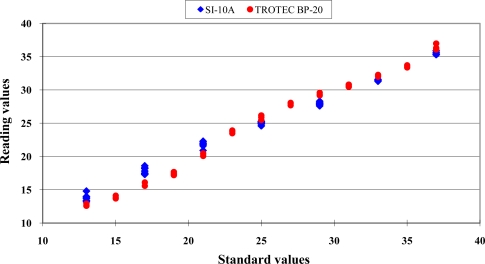
The relationship between the readout values of two types of infrared thermocouples *versus* standard values maintained by TC-2000 calibrator. Some data points are overlapped. Similar situations were found in the subsequent figures. 
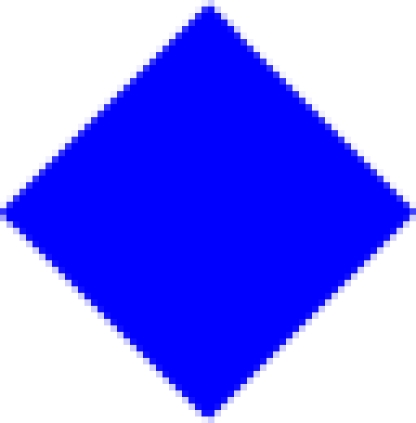
 Sentron SI-10A; and 
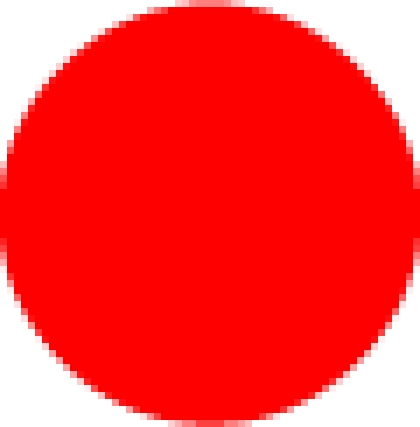
 Trotec BP-20.

The relationship between the reading values of the Sentron SI-10A infrared thermocouple versus standard values maintained by TC-2000 calibrator is shown in Figure 1.

**Figure 2.**
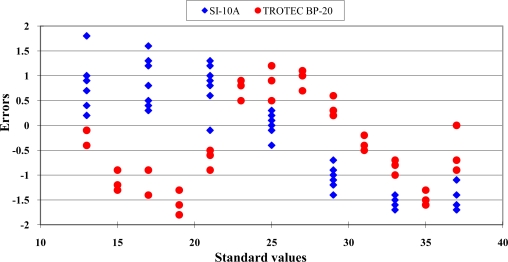
The error distribution of the Sentron SI-10A and Trotec BP-20 infrared thermocouple. “ 
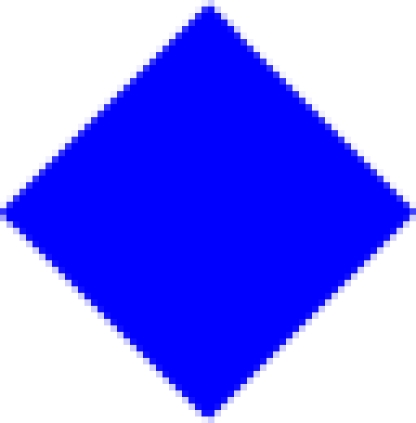
” is the symbol of the measurement data of SI-10A and “•” is the symbol of the measurement data of BP-20.

A nonlinear distribution of the data scattering was found. The error distribution of the Sentron SI-10A is shown in Figure 2.

**Figure 3.**
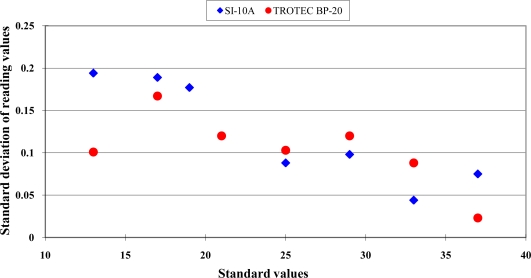
The standard deviations of the measured values at each standard environment for the Sentron SI-10A and Trotec BP-20 infrared thermocouple.

The errors ranged from −2.0 to 1.8 °C within the range from 13 °C to 21 °C. This revealed an over-estimation performance. When the measurement temperature was higher than 25 °C, the errors ranged from −0.6 to −1.8 °C. This represented an under-estimation performance. The readout values of the infrared thermocouples should not be recognized as the true values directly. The standard deviations of the measurement values at each standard environment are presented in Figure 3.

Below the measurement temperature of 25 °C, the standard deviations were less than 0.2 °C. In the higher temperature range, the standard deviations were less than 0.1 °C. From the viewpoint of practical applications, this sensor had good precision performance. The estimated parameters and statistics of the calibration equations for this Sentron SI-10A infrared thermocouple within the range from 13 °C to 37 °C are listed in Table 2.

A clear pattern or uniform distribution of residual plots could serve as a qualitative criterion to evaluate the calibration equation. If the data distribution of residual plots indicated a uniform distribution, the model could be recognized as adequate.

A clean systematic pattern of residual plots was found for linear and polynomial equations. However, a random distribution of residual plots was found for the three-order polynomial equation, indicating that the three-order polynomial equation was the adequate calibration equation for this sensor within the range from 13 °C to 37 °C. The standard deviation of estimators for the three calibration equations was 0.5541, 0.5410 and 0.3924 °C, respectively.

The nonlinear curves could be viewed as linear lines by narrowing the measurement range. As the measurement data within the narrower range were reevaluated, the calibration equations and statics of the Sentron SI-10A infrared thermocouple for the measurement data ranging from 20 °C to 35 °C are presented in Table 3. This range is the most common and useful for routine temperature estimation.

According to the statistical residual plots procedure, the linear calibration equation is recognized as the adequate model by the residual plots. A polynomial equation did not improve the predictive ability by the standard deviations *s* and coefficient of determination, *R^2^*. The numeric value of the standard deviation of estimated values was 0.374 °C. This indicates that the accuracy of this infrared thermocouple was improved by the calibration equation within a narrower measurement range.

### 3.2. Performance of the TROTEC BP-20 Sensor

The distribution between readout values of the Trotec BP-20 infrared thermocouple and the standard temperature are shown in Figure 1. A typical nonlinear data distribution was found. The error distribution is presented in Figure 2. The errors distribution showed a clear nonlinear pattern. The standard deviation of readout values at each standard temperature is presented in Figure 3. The good repeatability was confirmed for this sensor. The numeric values of standard deviations were less than 0.18 °C. The calibration equations and criteria are listed in Table 4.

Within the range from 13 °C to 37 °C, the adequate equation was a complex four-order polynomial equation. The standard deviation of the estimated values for the four calibration equations was 0.8661, 0.7584, 0.7355 and 0.3366 °C, respectively. The results indicated that the sensor required a high-order polynomial equation to decrease the predictive errors. Only the four-order equation had a uniform distribution of residual plots. As the measurement data were limited over a 20–35 °C range, the regression analysis results are presented in Table 5.

For the linear calibration equation, the standard deviation of estimated values was 0.8209 °C. A clear systematic pattern of residual plots was found. The two-order polynomial equation could be recognized as the adequate calibration equation by the residual plot. The predictive error was 0.3322 °C.

### 3.3. The Difference Performance between Sensors

**Figure 4.**
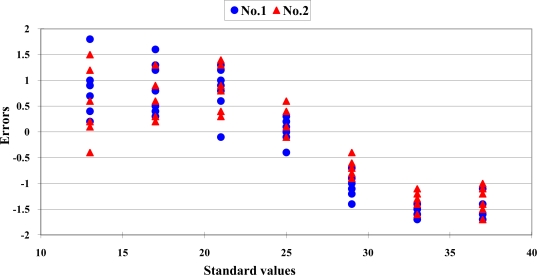
The errors distribution of two SENTRON SI-10A infrared thermocouples from the same manufacturer and production batch.

To evaluate the reproducibility of the calibration equations, the error distribution of two Sentron SI-10A infrared thermocouples from the same manufacturer and the same production batch are shown in Figure 4. The data distribution showed a significant difference between the two sensors. The calibration equation of No.1 sensor has been established. When the measurement data of sensor No. 2 were transformed with the calibration equation developed from sensor No.1, the predictive errors were higher than 1.0 °C.

**Figure 5.**
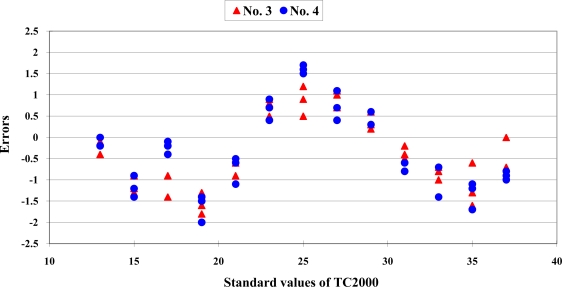
The errors distribution of two Trotec BP-20 infrared thermocouples from the same manufacturer and production batch.

The error distributions of two TROTEC BP-20 infrared thermocouples are presented in Figure 5. When the measurement data of sensor No. 4 was calculated with the calibration equation that was established from sensor No. 3, the predictive errors were higher than 1.2 °C.

From the above results, we can conclude that we must establish a special calibration equation for each infrared thermocouple to ensure the accuracy of its measurement performance.

The results of this study indicated that the accuracy of two-kinds of infrared thermocouples could be significantly improved using calibration equations. The form of the adequate calibration equation was influenced by the measurement range. For Sentron SI-10A sensors, a three-order polynomial equation was adequate for the measurement data over a 13–35 °C range. However, a linear calibration equation was valid for a narrower measurement range (20 °C–35 °C). Similar results have been reported on the manuals of some manufacturers [11,17]. For the Trotec BP-20 infrared thermocouple, the adequate calibration was a four-order polynomial equation for the measurement over a 13–37 °C range and a two-order polynomial equation for the measurement over a 20–35 °C range. Similar results were found as the research results of Bugbee *et al*. [8]. They selected the residual plots as a criterion to evaluate the calibration equations and the general calibration model for three types of infrared thermometer were two-order polynomial equations over a 15–35 °C range.

A linear calibration equation was proposed by Baker *et al*. for the measurement values of an Everest 4000A infrared thermometer and the standard temperature [9]. The coefficient of determination was close to unity and the mean absolute error was 0.028 °C. Similar results were reported by Savage and Heilman [10]. After finishing the laboratory test of twenty-one infrared thermometers, their report indicated that all the coefficients of determination were higher than 0.9965 and the *RMSE* value ranged from 0.11 °C to 1.10 °C. The adequateness of the linear equation was not checked. The coefficients of determination *R^2^* of the calibration equations presented in Tables 2, 3, 4 and 5 of this study were all near to unity. However, the residual plots showed that some equations cannot be recognized as adequate calibration equations, indicating that the coefficient of determination cannot be applied as the sole criterion to evaluate the calibration equation.

Mahan and Yeater [14] defined the infrared thermometer as the industrial quality infrared thermometer (IRT) and the infrared thermocouple as the consumer quality IRT. In their study, a question “can a low-cost consumer quality IRT replace an expensive industrial quality IRT for the applications in agriculture?” was addressed. From the above discussions, the accuracy of the infrared thermometer ranged from 0.03 °C to 1.1 °C [6,8–10]. The results of this study indicated that the errors of the readout values without using calibration equations for the two kinds of infrared thermocouple ranged from −1.8 °C to 1.8 °C. However, the accuracy of these infrared thermocouples could be maintained at nearly 0.4 °C after improvement by appropriate calibrations.

In the study of the estimation of stomatal conductance, the difference between canopy and air temperature was below 0.8 °C [2]. The difference between canopy temperature and air temperature (canopy- air temperature) could serve as crop water stress index. The canopy- air temperature range was from −2 °C to 9 °C according to the study of Gontia and Tiwari [4], from −3 °C to 3 °C in the study of Abraham *et al*. [18] and from −5 °C to 12 °C in the study of Al-Faraj *et al*. [19]. The accuracy of the infrared thermocouple in this study was nearly 0.4 °C after using the calibration equations. The performance test of this study was executed in the laboratory. The effects of the influencing factors on the performance in the field test need to be further studied and the measurement uncertainty of this sensor needs to be evaluated under those conditions. Considering the required performance of temperature measurement, the infrared thermocouple could not be applied to calculate the crop water stress index in this stage. The use of an infrared thermometer for *in situ* and real time measurements of the leaf temperature has been recommended [20]. This infrared thermocouple could serve as a mobile and useful tool to measure the leaf temperature. In this study, the performance of two kinds of infrared thermocouples was evaluated. A standard operation procedure (SOP) for infrared thermocouple performance evaluation is proposed in the following section. This SOP could be applied for other infrared thermocouples.

### 3.4. The Standard Operation Procedure (SOP) of Infrared Thermocouple Performance Evaluation

#### 3.4.1. Standard Temperature

The standard temperature of the black source is maintained by a temperature calibrator and a blackbody source. The uncertainty of the temperature calibrator is within 0.03 °C. The emittity of the blackbody source is nearly 0.9999.

#### 3.4.2. Test Environment

The test environment was maintained at 25 °C and the variation of the setting room environment temperature was controlled within ± 1.5 °C.

#### 3.4.3. Test Procedure

- The target temperature for calibration was maintained at different range according the practical requirement. The interval of testing temperature is 2 °C.
- Three or more replicates are made on each measurement point.
- As one measurement is finished, the infrared thermocouple was taken out from the blackbody cavity for five minutes and then was put back into the cavity for further measurements.

#### 3.4.4. Data Analysis

The performance of these infrared thermocouples was assessed by their accuracy and precision. The accuracy is expressed as the closeness with which a measurement value approached to the standard value. The error *e_i_* is defined as the difference between the readout value of the infrared thermocouple and the standard temperature. The precision *P* is expressed as the repeatability of these measurement values in the same standard environment. The precision was calculated as the estimated deviation of *e_i_* values. A smaller *P* value revealed better precision performance.

#### 3.4.5. The Calibration Equation

The calibration equations were established using the regression analysis technique. The criteria for selecting of the best equation are the coefficient of determination *R^2^*, the standard deviations of estimator *s*, t-test of each parameters and residual plots. The inverse calibration polynomial equation was adopted.

## 4. Conclusions

In this study, the performance of two-kinds of infrared thermocouples was evaluated. The temperature maintained by the temperature calibrator and a specific black cavity served as the standard temperature. As the reading values of these sensors were used directly as true values, the error distributions ranged from −1.8 °C to 1.8 °C. The accuracy of these sensors could be maintained at nearly 0.4 °C by the calibration equations. The form of the adequate calibration equations was different due to the different manufacturers of infrared thermocouple and the measurement range. Compared with the industrial level infrared thermometer, the main advantage of infrared thermocouples is their cost. From the performance evaluation, these sensors could be applied to *in situ* and real time measurement of leaf temperatures for the management of forest or agricultural resources. Future studies will include field tests and the calculation of the measurement uncertainty.

## Figures and Tables

**Table 1.** Specifications of the thermopile infrared thermometers.

Parameters	Sentron SI-10A	Trotec BP-20
Operating temperature	0 to 50 °C	0 to 50 °C
Accuracy	±1.0 °C (15–35 °C)	±1.0 °C (21 °C to 200 °C)
Resolution	0.1 °C	0.1 °C
Field of view	11 to 1	12 to 1
Response time	0.1 sec	0.3 sec
Wavelength	5 ∼ 14 μm	6 ∼ 14 μm
Signal indication	LCD screen	LCD screen

**Table 2.** The calibration equations and statistics of the SENTRON SI-10A infrared thermocouple, measurement ranged from 13 °C to 37 °C.

**Form**	**Equation**	**R^2^**	**S**	**Residual plots**
Linear	T_s_ = −3.0932 + 1.1336 T_r_	0.9954	0.5541	Clear pattern ^a^
Polynomial	T_s_ = −1.4215 + 0.9858 T_r_ + 0.003 T_r_^2^	0.9980	0.5410	Clear pattern
Third-order	T_s_ = −16.0685–1.3945T_r_ +0.1045T_r_^2^ − 0.0014 T_r_^3^	0.9999	0.3924	Uniform ^b^

a Clear pattern: the residual plots revealed the systematic clear pattern;

b Uniform: the residual plots revealed uniform distribution.

**Table 3.** The calibration equations and statistics of the SENTRON SI-10A infrared thermocouple within the narrower measurement ranged from 20 °C to 35 °C.

**Form**	**Equation**	**R^2^**	**s**	**Residual plots**
Linear	T_s_ = −6.2284 + 1.2514 T_r_	0.9935	0.374	Uniform
Polynomial l	T_s_ = −7.2385 + 1.3287 T_r_ − 0.00145 T_r_^2^	0.9940	0.371	Uniform

**Table 4.** Calibration equations and statistics of TROTEC BP-20 infrared thermocouple within the ranged from 13 °C to 37 °C.

**Form**	**Equation**	**R^2^**	**s**	**Residual plots**
Linear	T_s_ = 0.8999 + 0.9789 T_r_	0.9936	0.8661	Clear pattern
Polynomial	T_s_ = 5.6083 + 0.5440 T_r_ + 0.009037 T_r_^2^	0.9953	0.7584	Clear pattern
Third-order	T_s_ = −3.0308 + 1.7307 T_r_ − 0.04161 T_r_^2^ + 0.000682 T_r_^3^	0.9956	0.7355	Clear pattern
Four-order	T_s_ = −95.6884 + 18.8478 T_r_ − 1.1713 T_r_^2^ + 0.03238 T_r_^3^ − 0.0003204 T_r_^4^	0.9991	0.3366	Uniform

**Table 5.** The calibration equations and statistics of TROTEC BP-20 infrared thermocouple within the ranged from 20 ° to 35 °C.

**Form**	**Equation**	**R^2^**	**s**	**Residual plots**
Linear	T_s_ = −2.3181 + 1.08489 T_r_	0.9852	0.8209	Clear pattern
Polynomial	T_s_ = 29.7702 − 1.3499 T_r_ + 0.04504 T_r_^2^	0.9986	0.3332	Uniform
